# Tracheobronchial involvement of mantle cell lymphoma

**DOI:** 10.1002/rcr2.346

**Published:** 2018-07-11

**Authors:** Lin Tong, Guixiang Gan, Chen Xu, Ling Yuan, Zhuozhe Li, Huayin Li

**Affiliations:** ^1^ Department of Pulmonary Medicine Zhongshan Hospital, Fudan University Shanghai China; ^2^ Shanghai Respiratory Research Institute Shanghai China; ^3^ Department of Pulmonary Medicine Hunan Provincial People's Hospital/The First Affiliated Hospital of Hunan Normal University Changsha China; ^4^ Department of Pathology Zhongshan Hospital, Fudan University Shanghai China; ^5^ Department of Hematology Zhongshan Hospital, Fudan University Shanghai China

**Keywords:** Bronchoscopy, mantle cell lymphoma, tracheobronchial wall thickness

## Abstract

Mantle cell lymphoma (MCL) is a rare type of B‐cell non‐Hodgkin’s lymphoma that commonly affects extranodal sites; however, tracheobronchial involvement is rare. We report the case of a 65‐year‐old male who presented with cough and dyspnoea. A chest computed tomography (CT) revealed irregular wall thickening of the trachea and bilateral bronchi and bilateral bronchiectasis. A bronchoscopy revealed a diffuse irregular surface of the tracheal and bilateral bronchial mucosa and polyposis‐like lesions. He was diagnosed with MCL based on an endobronchial biopsy, and then, the diagnosis was confirmed with a biopsy of the fluorodeoxyglucose (FDG)‐avid nasal mucosal soft tissue.

## Introduction

Mantle cell lymphoma (MCL) is a distinct type of B‐cell non‐Hodgkin’s lymphoma, which is characterized by t(11;14)(q13;q32) and Cyclin D1 over‐expression. Mantle cell lymphoma represents about 3%–10% of all non‐Hodgkin’s lymphomas. Most patients are in their 60s at the time of diagnosis, with a male predominance, and they often present with advanced stage disease (stages III–IV), frequently involving multiple extranodal sites, such as the gastrointestinal tract, spleen, bone marrow, liver, Waldeyer’s ring, skin, lacrimal glands, and central nervous system. Despite the frequency of extranodal disease in MCL, it is still uncommon for the airway of the lung to be involved 1, 2, 3. We report a case of MCL with trachea and bilateral bronchi involvement, and the diagnosis was confirmed by endobronchial biopsy with bronchoscopy.

## Case Report

**Figure 1:**
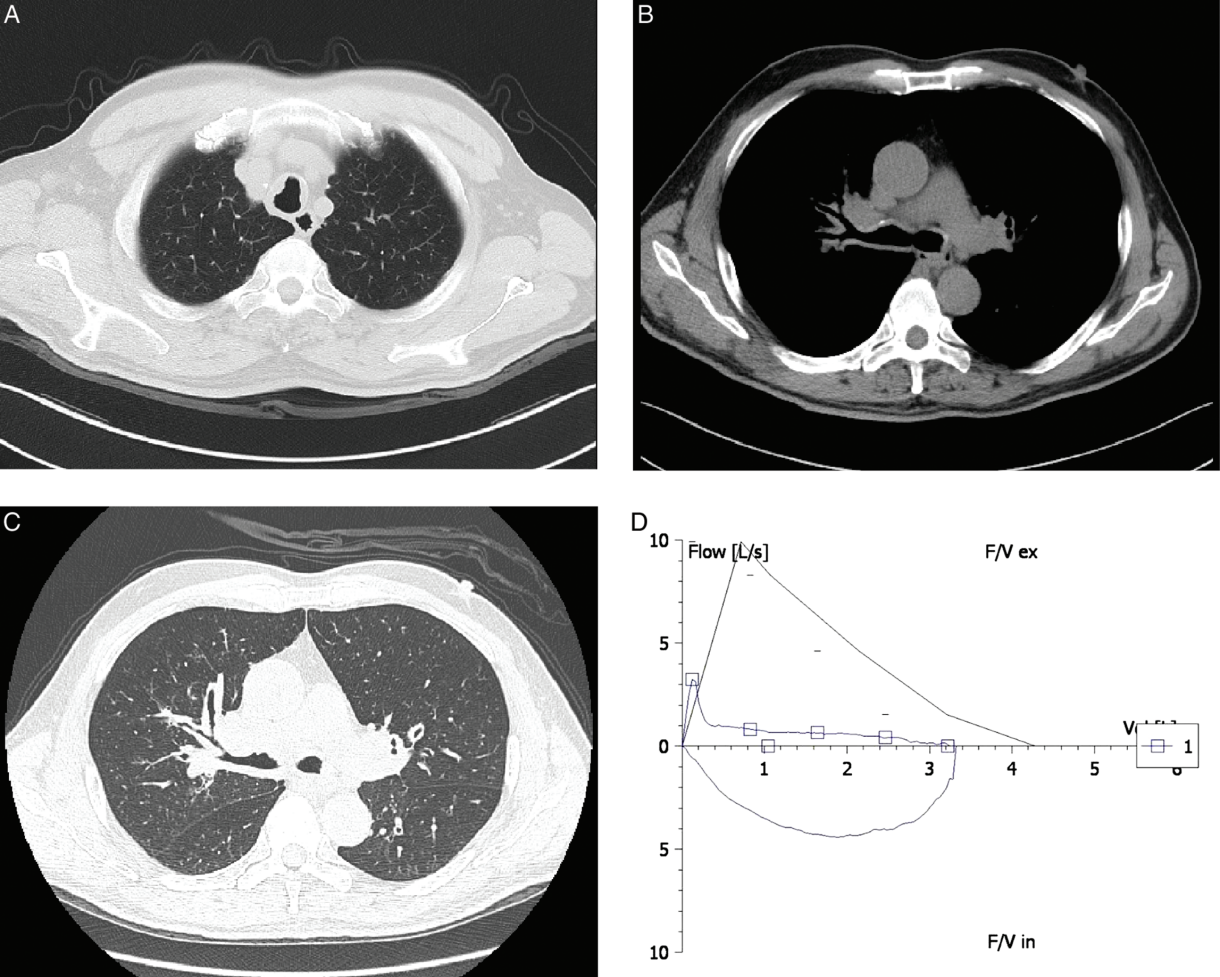
Non‐contrast chest computed tomography scan showing irregular tracheal wall thickening (A) and bilateral bronchial wall thickening (B, C). Pulmonary function testing showing obstructive ventilatory dysfunction (D).

**Figure 2:**
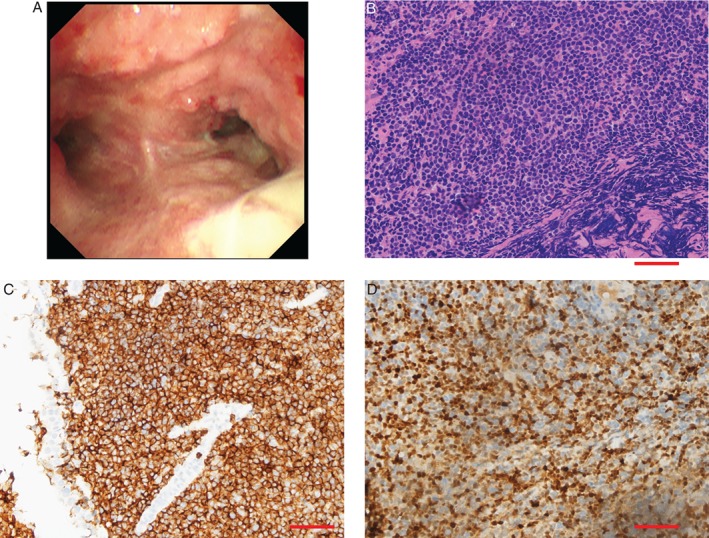
Bronchoscopy revealed a diffuse irregular surface of the tracheal and bilateral bronchial mucosa and multiple macroscopic submucosal nodules involving the trachea, the distal trachea above the major carina, and throughout the left and right main bronchi (A). Endobronchial biopsies of the mucosa on the major carina showed a population of atypical lymphocytes (B, H&E staining, original magnification ×400, bar = 10 μm). Immunohistochemistry staining showed that these lymphocytes were positive for CD5 (C) and cyclin D1 D (original magnification ×400, bar = 10 μm).

Our patient is a 65‐year‐old male who presented with a two‐year history of productive cough and progressive dyspnoea. He had no prior medical history and was a smoker of 40 pack‐years. On physical exam, he had mild fine bibasilar crackles on lung exam without wheezing. His laboratory values were all normal, with the exception of his arterial blood gas analysis, which showed a lower PaO_2_ of 74.4 mmHg on room air. His serum soluble interleukin‐2 receptor (sIL‐2R) level was high (1655 U/mL). Pulmonary function testing revealed severe irreversible obstructive ventilatory dysfunction with normal diffusing capacity of the lung (Fig. 1). A computed tomography (CT) scan of the chest revealed irregular wall thickening of the trachea and bilateral bronchi (Fig. 1). In addition, there was bilateral bronchiectasis, and mediastinal and both hilar lymphadenopathy were noted. Flexible bronchoscopy demonstrated a diffuse irregular surface of the tracheal and bilateral bronchial mucosa and multiple macroscopic submucosal nodules involving the trachea, the distal trachea above the major carina, and throughout the left and right main bronchi (Fig. 2). Endobronchial biopsy of the mucosa on the major carina showed a population of small atypical lymphocytes with scant cytoplasm and hyperchromatic nuclei of irregular nuclear contours. These atypical lymphocytes were CD20 positive B‐cells co‐expressing CD5, blc‐2, and Cyclin D1, leading to the diagnosis of MCL (Fig. 2). Further positron emission tomography (PET)‐CT imaging showed the persistence of FDG‐avid lymphadenopathy within the chest (SUVmax 4.2) and FDG‐avid wall thickening of the trachea and bilateral main bronchi (SUVmax 2.8), as well as FDG‐avid nasopharyngeal soft tissue thickening (SUVmax 6.8). Biopsies of the nasopharyngeal mucosa further demonstrated B‐cells with the same immunophenotype as the endobronchial biopsy, and 11;14 translocation was confirmed by fluorescent in situ hybridization (FISH) analysis using a CCND1/IGH probe. Bone marrow biopsy showed an increased lymphocyte percentage of 23%, with 1% of immature lymphocytes, and a positive immunoglobulin rearrangement (FR1‐JH(+), FR2‐JH(+), FR3‐JH(+), Vk‐Jk(+), Vk‐Kde + intronKde(−)). Bone marrow flow cytometry demonstrated a surface kappa clonal B‐cell population with CD5, CD20, and FMC‐7 expression. Taken together, these findings were consistent with an MCL involving the tracheobronchial wall, nasopharyngeal soft tissue, and bone marrow.

## Discussion

Mantle cell lymphoma is a mature B‐cell non‐Hodgkin’s lymphoma characterized by the proliferation of B‐cells resembling those found in the follicular mantle zones, a subset arising from antigen‐experienced B cells. Tumour cells are typically CD5, CD19, and CD20 positive, along with CD10 and CD23 negative. The vast majority over‐expresses Cyclin D1, which is not typically expressed in normal lymphocytes. Cyclin D1 over‐expression is strongly associated with the translocation between the CCND1 gene on chromosome 11 and the IGH gene on chromosome 14 [t(11;14)(q13;q32)]; however, this translocation is present by karyotyping in only 50%–65% of patients 3. Secondary genetic events increase the oncogenic potential of Cyclin D1 and frequently inactivate DNA damage response pathways. However, Cyclin D1‐negative cases with a typical morphology and gene expression profile have been described and often show over‐expression of Cyclin D2 or D3. Recently, SOX11 has been described as a diagnostic maker that is equally expressed in D1‐positive and D1‐negative MCL 3. The histological pattern of MCL may be diffuse, nodular, mantle zone, or a combination of the three patterns of growth. Some reports indicate a better prognosis for cases with a mantle zone pattern. Despite the frequency of extranodal disease in MCL, it is still distinctly uncommon for the lung parenchyma to be involved 1.

Radiographic features of pulmonary lymphoma on chest CT imaging are varied but classically present in one of three patterns. The most common appearance is that of scattered ill‐defined nodules with lower lobe predominance. The second most common pattern is a bronchovascular/lymphangitic process with coarse linear and reticulonodular shadows extending outwards from the hilum in a perivascular and peribronchial distribution. The third pattern is described as pneumonic/alveolar and is effectively indistinguishable from bacterial pneumonia 1. However, airway obstruction caused by the massive endobronchial growth of lymphoma cells is a very rare presentation of pulmonary lymphoma. It is well recognized that airway obstruction can be due to primary tracheal lymphoma or tracheobronchial compression by enlarged nodal lymphoma. In the present patient, pulmonary function testing indicated severe irreversible airway obstruction, and further pathological analysis demonstrated that bilateral bronchial occlusion was caused by the massive endobronchial growth of lymphoma cells. Endobronchial non‐Hodgkin’s lymphoma is extremely rare and usually occurs in the presence of disseminated disease. Bronchoscopic examination with biopsy is essential for the prompt diagnosis of this condition. Furthermore, PET/CT has a high sensitivity in detecting both nodal and extranodal disease in patients with malignant lymphoma 4, 5. The present patient underwent PET/CT, which revealed the involvement of lymph node within the chest, the tracheobronchial wall, and the nasopharynx. To the best of our knowledge, only four cases of MCL with tracheobronchial involvement have been previously reported (Table 1) 6, 7, 8, 9. Endobronchial involvement of non‐Hodgkin’s lymphoma is categorized into two patterns: diffuse submucosal infiltration (type I) and localized solitary mass (type II) 10. The previous four cases and the present case all exhibited a type I pattern.

**Table 1:** Tracheobronchial involvement of mantle cell lymphoma.

References	Age	Gender	Duration between first diagnosis of MCL and tracheobronchial involvement	Smoking history	Presenting symptoms	Cyclin D1	t(11;14) (q13;q32)
Figgis et al. 6	53	Female	More than three years (second relapse)	N/A	Cough, dyspnoea, wheeze	N/A	N/A
Miyoshi et al. 7	70	Female	Five years (fifth relapse)	N/A	Stridor, respiratory failure	Positive	Negative
Imai et al. 8	86	Male	Two years (first relapse)	N/A	Dyspnoea	N/A	N/A
Katono et al. 9	87	Male	0 (diagnosed by endobronchial biopsy)	Never‐smoker	Dyspnoea on exertion	Positive	N/A
Current case	65	Male	0 (diagnosed by endobronchial biopsy)	Current smoker with 40 pack‐years	Productive cough, dyspnoea	Positive	Positive

MCL, mantle cell lymphoma; N/A, not available.

Mantle cell lymphoma is one of the most difficult‐to‐treat B‐cell lymphomas. Although conventional chemotherapy induces high remission rates in previously untreated patients, relapse within a few years is common, contributing to a rather short median survival of five to seven years 3, 11. In addition to conventional chemotherapy, there are three classes of novel agents available for the treatment of relapsed MCL patients, namely, proteasome inhibitors, mTOR inhibitors, and lenalidomide, although we still have only a limited understanding of their mechanisms of action and determinants of efficacy 3. Thus, patients with MCL should be encouraged to enrol in clinical trials with a strong translational research programme.

In conclusion, the tracheobronchial involvement of MCL is rare. Bronchoscopic findings revealed a diffuse irregular surface of the tracheal and bilateral bronchial mucosa and polyposis‐like lesions. A pathological diagnosis can be made based on an endobronchial biopsy. Tracheobronchial wall findings on chest CT should be carefully examined in patients with obstructive ventilatory dysfunction on pulmonary function testing and should prompt efforts to establish a tissue diagnosis if necessary.

### Disclosure Statement

Appropriate written informed consent was obtained for publication of this case report and accompanying images.
